# Accuracy and patient satisfaction of intraoral scanner in preschool-aged children: a combined in vivo and in vitro study

**DOI:** 10.1186/s12903-025-07406-z

**Published:** 2025-11-28

**Authors:** Jie Peng, Huan Zeng, Yang Liao, Hongmei Zhang

**Affiliations:** 1 The Affiliated Stomatological Hospital of Chongqing Medical University, Chongqing, 401147 China; 2 Chongqing Key Laboratory of Oral Diseases, Chongqing, 401147 China; 3 Chongqing Municipal Key Laboratory of Oral Biomedical Engineering of Higher Education, Chongqing, 401147 China; 4 Chongqing Municipal Health Commission Key Laboratory of Oral Biomedical Engineering, Chongqing, 401147 China

**Keywords:** Intraoral scanner, Primary dentition, Impression accuracy, Patient comfort

## Abstract

**Objectives:**

This study aims to evaluate the accuracy of intraoral scanner (IOS) in both intraoral and extraoral settings within primary dentition, as well as patient satisfaction among preschool-aged children.

**Methods:**

Forty-one preschool-aged children underwent intraoral scanning with the iTero Element 2 scanner and conventional alginate impressions (CI). Resulting plaster models were digitized via both IOS and a desktop scanner. To assess precision, one plaster model was scanned ten times with the IOS. Accuracy was measured via Geomagic Control 2022, which evaluated root mean square (RMS) deviation and intra-arch linear distances. A validated questionnaire was used to access the degree of comfort and preference between IOS and CI. Statistical analyses included paired t-tests, repeated-measures ANOVA, Friedman tests and Wilcoxon signed-rank tests, with statistical significance set at *P* < 0.05.

**Results:**

The trueness of the intraoral scan was 0.1282 ± 0.0230 mm (maxilla) and 0.1341 ± 0.0255 mm (mandible), whereas the extraoral scan showed significantly higher trueness (0.0399 ± 0.0059 mm in the maxilla, 0.0430 ± 0.0087 mm in the mandible) (*P* < 0.001). The precision was 0.0347 ± 0.0066 mm for the maxilla and 0.0358 ± 0.0063 mm for the mandible. Most intra-arch distances showed no significant variation across scanning methods. The questionnaire responses favored IOS, with significantly greater comfort and preference (*P* = 0.014), despite some perceptions of scanner bulkiness.

**Conclusions:**

The IOS delivers high accuracy for both intraoral and extraoral scans in primary dentition and is well accepted by preschool children, showing potential for wider application in pediatric dentistry.

**Supplementary Information:**

The online version contains supplementary material available at 10.1186/s12903-025-07406-z.

## Background

In pediatric dentistry, accurate replication of dental arches is crucial for clinical procedures such as the fabrication of crowns, space maintainers, and early orthodontic intervention. Conventional impression (CI) of elastomeric materials has been considered the gold standard for dental models [1], while alginate impression is favored for cost-efficiency, ease of use, rapid setting time, and simplicity of technique [2]. Despite these advantages, CI is frequently associated with patient discomfort, gag reflex, and anxiety, particularly in patients with heightened sensitivity [3–6].

The introduction of intraoral scanners (IOS) has significantly transformed dental workflow by enabling direct digital impressions [7]. This technology offers several advantages, including convenient storage, intuitive communication, biological safety, and the prevention of damage during model transportation [8]– [9]. To minimize the risk of damage during transportation and reduce shipping costs, IOS is occasionally employed to scan plaster models. While IOS has become integral in pediatric dentistry, many clinicians still depend on plaster casts. As the need to store plaster casts in digital format grows, direct scanning of these casts with IOS becomes essential for clinicians [10]. In this context, assessing the reproducibility of ex-vivo scans, alongside in-vivo scans, holds considerable value for clinical practice. Numerous in vivo and in vitro studies have confirmed that IOS meets the clinical accuracy requirements for full-coverage restorations and orthodontic treatments in both mixed and permanent dentitions [11–14]. However, several device-related factors, patient-related factors and operators’ experience influence the accuracy of IOS [15]– [16]. Device-related factors include the scanning pattern [17], ambient lighting [18], temperature, calibration [19], and scanning head size [20]. Patient-related factors include the presence of blood, saliva, or gingival fluid [21–23], tooth type [24], and arch width [25]. Recent studies suggest that both adults and adolescents prefer IOS because of its efficiency and non-invasiveness [14, 26]. However, there is a lack of research on the application of IOS in the primary dentition. Compared with permanent dentition, primary dentition has unique tooth morphology characteristics and physiological spaces. Moreover, preschool-aged children pose a set of challenges including increased oral sensitivity, smaller anatomical dimensions, and unpredictable behavior, all of which may affect both scan accuracy and patient comfort.

As digital workflow is becoming more prevalent in pediatric dentistry, it is essential to assess both the accuracy of IOS in primary dentition and the comfort of preschool-aged children during IOS and CI procedures. Therefore, we propose the following null hypotheses:

1. No significant difference in accuracy is detected among the intraoral scans of iTero element 2, extraoral scans, and CI in primary dentition.
2. Preschool-aged children perceive no difference in comfort between IOS and CI techniques.

## Materials and methods

### Study design

This single-center study was conducted following the Declaration of Helsinki and was approved by the Institutional Review Board of the Affiliated Stomatological Hospital of Chongqing Medical University (Approval No: 2024174). Written informed consent was obtained from legal guardians, and verbal assent was obtained from participating children. Eligible participants were 3 to 6 years old, had complete primary dentition, good oral hygiene, no prior impression experience, and were able to cooperate during procedures. Psychiatric comorbidities or developmental disorders were ruled out based on past medical history and clinical examination. All participants were enrolled from the Pediatric Dentistry Department from March 2024 to March 2025.

### Data collection

The sample size for this repeated-measures accuracy study was calculated using G*Power software (version 3.1). A power analysis (F-tests, Repeated-measures ANOVA, within factors) was conducted with the following parameters: power (1-β) = 0.95, α = 0.05, effect size *f* = 0.864 (as derived from the study by Qian X, 2023 [27]), number of groups = 3, and correlation among repeated measures = 0.8. The calculation indicated that a minimum of 15 participants would be required. The sample size of the questionnaire should be at least five times the number of questions. Intraoral scans were performed with an iTero Element 2 scanner (v1.13.25.70, Align Technology, USA, official accuracy is 0.02 mm). Alginate impressions (Dentsply GAC, USA) were taken via standardized trays and dental stone (Heraeus, Germany) was added. Then plaster models were subsequently scanned via both IOS and the 3 Shape desktop scanner (E4, 3 Shape, Denmark) in extraoral setting. Repeated in vivo scans were not possible due to the limited tolerance of preschool-aged participants. For precision assessment, ten repeated extraoral scans via IOS were conducted on a single randomly selected plaster model. Following each impression procedure, the subjects’ attitudes and perceptions were immediately assessed. The procedures were performed strictly according to the manufacturers’ protocols and standardized operating procedures by the same examiner (J.P.), who has extensive experience in professional scanning training and three years of clinical scanning practice. The IOS scans were performed in a specific sequence: beginning with the lower jaw, followed by the upper jaw, and finally the inter-occlusal relationship. The process starts with the occlusal surfaces, then moves to the lingual and palatal surfaces, continues with the buccal surfaces, and concludes by filling in any remaining areas. Throughout the scanning process, the scanner head was maintained in close contact with the tooth surfaces. When scanning the buccal and lingual surfaces, the scanner head was positioned at a 45-degree angle to the tooth surface to ensure optimal accuracy. The scanners were automatically calibrated before each scan.

### Digital model processing and comparative analysis

All digital models were exported in standard tessellation language (STL) format. Scans obtained from the desktop scanner were designated the control group (C), intraoral scans designated the test group (T), and extraoral scans acquired via IOS were assigned to the expansion group (E). Geomagic Control 2022 (USA) was used for analysis. For each T and E group model, the lowest points of the buccal and lingual gingival margins were selected as anatomical landmarks. Soft tissue beyond the dental arch was removed via the “Multiview Selection Function” while preserving the dental morphology and interdental papillae. The root mean square (RMS) was calculated according to a standardized three-stage alignment protocol: initial rough alignment with “N-point Registration” followed by “Global Registration” for refinement, and finalization with “Best-Fit Alignment” for optimal superimposition. Trueness was quantified RMS values and intra-arch distances with “Linear Measurement”, which included full dentition width (FDW), measured between the central pits of the second primary molars; anterior dentition width (ADW), measured between the cusp tips of the canines; anterior dentition length (ADL) and full dentition length (FDL), both defined as the linear distance from the contact point of the central incisors to the respective transverse dimension. The RMS was also used to quantify the precision of intraoral scanning.

### Reliability assessment

**Fig. 1:**
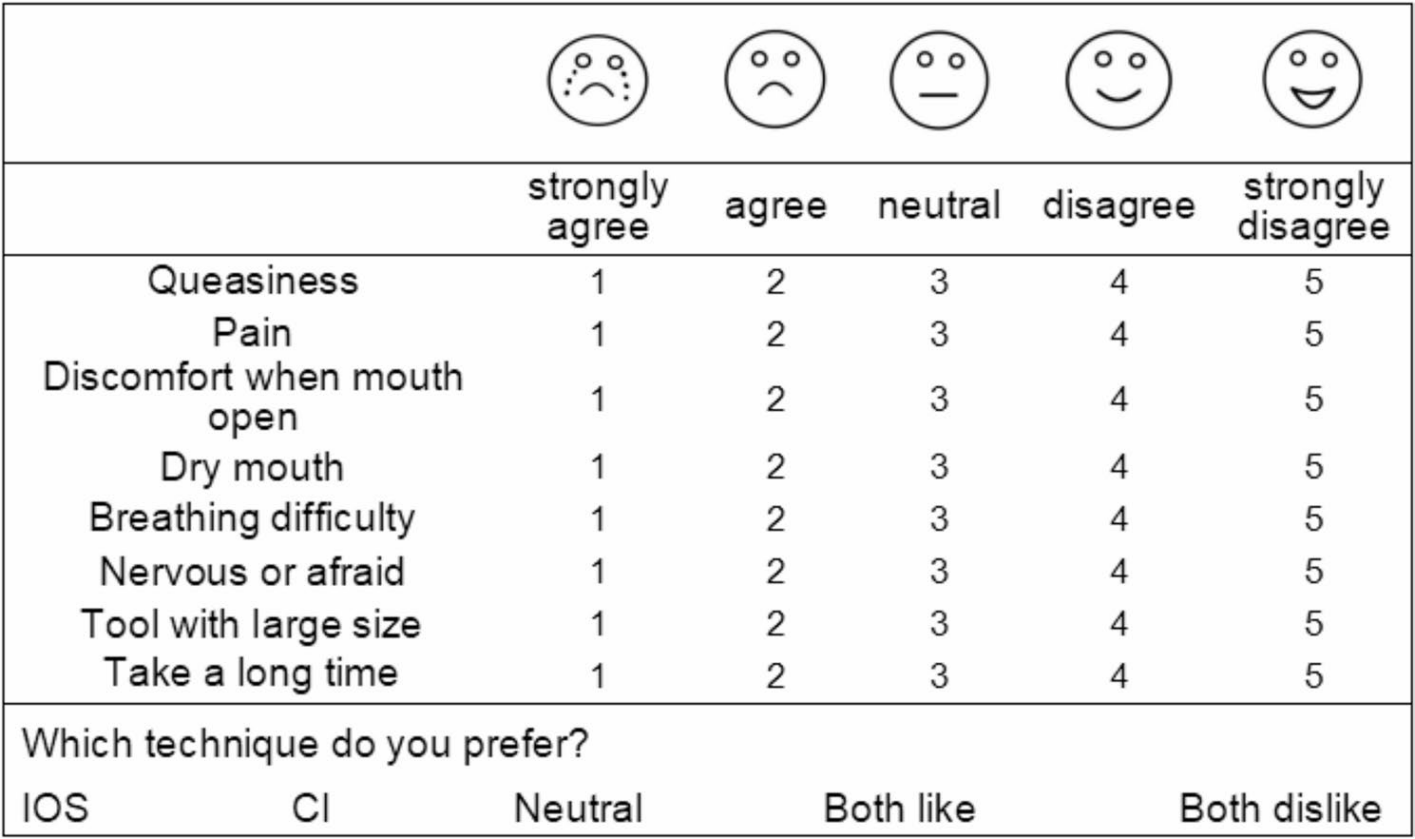
Perception questionnaire. IOS: Intraoral scanner; CI: Conventional impression

The questionnaire was pre-tested and revised before use. A pilot test was conducted on 18 preschool-aged children who had alginate impressions or digital models for orthodontic purposes. The final version included eight 5-point Likert scale questions and one preference question (Fig. 1). These questions were designed to align closely with children’s typical experiences during dental impression procedures, while also being appropriately framed for their level of comprehension and prior validation through pre-testing to ensure high completion rates. It was noteworthy that previous studies [3, 5]– [6] have demonstrated the effectiveness of these questions in reliably capturing children’s perceptions of various impression methods. During impression taking, researchers carefully observed subtle behavioral responses, such as sudden frowning, crying, or refusal to open the mouth, to assist in accurate questionnaire completion. Furthermore, guardians were involved in the process to help explain the questions and facilitate communication, thereby improving child engagement and response quality. The internal consistency was acceptable (Cronbach’s α = 0.789).

The desktop scanner was tested with ten repeated scans, showing high precision with a mean deviation of 0.0169 ± 0.0003 mm. To evaluate the stability and reliability of the digital model measurement method, inter-observer and intra-observer reliability were assessed. Three blinded observers with varying experience levels - H.Z. (10 years), J.P., and Y.L. (both 3 years) - performed the RMS and intra-arch measurements on ten digital models from each of the three study groups. One researcher (J.P.) conducted repeated measurements over ten consecutive days. Reliability was assessed via the intraclass correlation coefficient (ICC) with a two-way random effects model, with both intra- and inter-rater reliability showing high consistency (ICC > 0.85) (Supplyment Table 1).

### Statistical analysis

Normality was assessed via the Shapiro-Wilk test. Parametric data (RMS values) were analyzed via paired t-tests. For dental arch measurements that followed a normal distribution, repeated-measures ANOVA with Bonferroni-adjusted pairwise comparisons was employed, while nonparametric data were evaluated using the Friedman test, followed by post hoc Wilcoxon signed-rank tests with Bonferroni correction for multiple comparisons. The questionnaire response was analyzed via nonparametric tests: the Wilcoxon signed-rank test for paired comparisons, the Mann-Whitney U test for independent groups, and the Kruskal-Wallis test for multiple groups. Preference data between IOS and CI were compared using Wilson score intervals to calculate 95% confidence intervals. All tests were two-tailed, with statistical significance set at *P* < 0.05. Analyses were performed via SPSS 27.0 (IBM Corp., Armonk, NY, USA).

## Results

A total of 41 children (12 boys, 29 girls) with a mean age of 3.8 years (range: 3–6 years) were initially enrolled in this study. The age distribution was as follows: 12 children were 3 years old, 25 children were 4 years old, 3 children were 5 years old, and 1 child was 6 years old. Intraoral digital models, plaster models, and questionnaire responses were successfully collected from all participants, though one plaster model was damaged during transportation, reducing the analyzable sample size for digital model accuracy to 40 cases. The post-hoc test statistical power is 1.0.

**Fig. 2:**
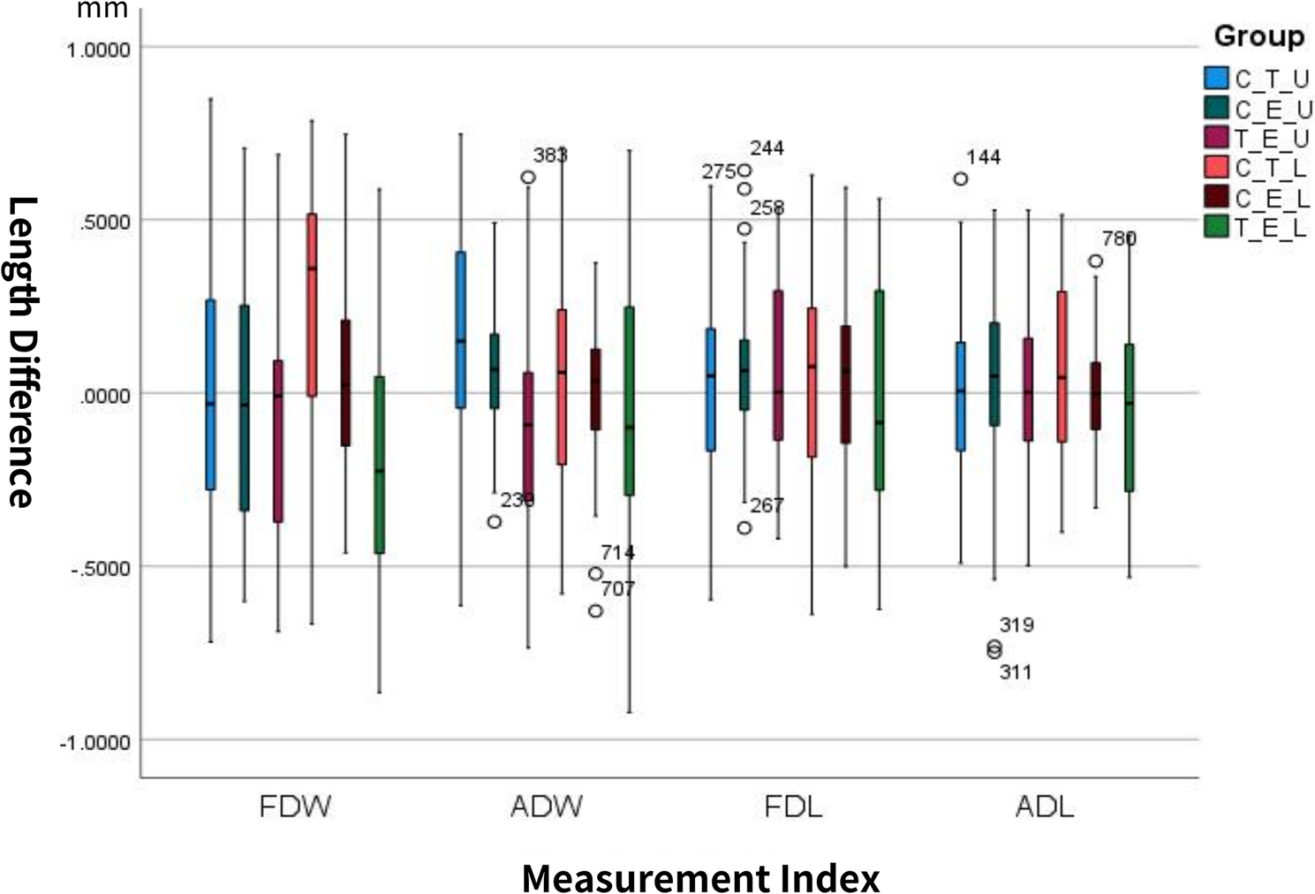
Box-and-whisker plots depicting the difference in intra-arch distances for the three groups. FDW: Full dentition width; ADW: Anterior dentition width; FDL: Full dentition length; ADL: Anterior dentition length; C: Control group; T: Test group; E: Expansion group; U: Upper dentition; L: Lower dentition

Comparative analysis revealed no significant RMS differences between mandibular and maxillary arches for either the T group (*P* = 0.258) or the E group (*P* = 0.069). However, the E group had significantly lower RMS values than the T group (*P* < 0.001) (Table 1). As shown in the box-and-whisker plots in Fig. 2, the differences in intra-arch distances among the three groups were generally distributed near zero. Significant differences were detected for the maxillary ADW (*P* = 0.005) and mandibular FDW (*P* < 0.001) (Table 2). Post-hoc analysis revealed that the maxillary ADW in T group were shorter than the C group (mean difference: 0.2007 mm; Cohen’s d = 0.085; *P* = 0.005). In addition, the mandibular FDW in the T group was shorter than that in the C group (mean difference: 0.3083 mm; Cohen’s d = 0.169; *P* < 0.001) and was also shorter than that in the E group (mean difference: 0.2536 mm; Cohen’s d = 0.139; *P* < 0.001). The IOS used in an extraoral setting exhibited a high level of precision for both the maxillary and mandibular arches, with no significant difference between them (maxilla: 0.0347 ± 0.0066 mm; mandible: 0.0358 ± 0.0063 mm, *P* = 0.806).

**Table 1:** RMS deviation of trueness for intraoral and extraoral scanning (mm)

		**Min**	**Max**	**Mean**	**SD**	**Shapiro–Wilk *P*Value**	**t**	***P***
Maxilla	T	0.0831	0.1750	0.1282	0.0230	0.135	14.377	<0.001***
	E	0.0286	0.0504	0.0399	0.0059	0.335		
Mandible	T	0.0638	0.1832	0.1341	0.0255	0.060	13.430	<0.001***
	E	0.0304	0.0661	0.0430	0.0087	0.089		

*RMS* Root Mean Square, *T* Test group, *E* Expansion group, *SD* Standard deviation, *t* t value of paired t test

***Statistical significance at P ≤ 0.001

**Table 2:** The results of the Friedman test and post-hoc tests for intra-arch distances

		***P***	**Sig. bet. periods**		
			***P1***	***P2***	***P3***
Maxilla	FDW	0.732	NS	NS	NS
	ADW	0.001***	0.005**	NS	NS
	FDL	0.461	NS	NS	NS
	ADL	0.407	NS	NS	NS
Mandible	FDW	<0.001***	<0.001***	NS	<0.001***
	ADW	0.405	NS	NS	NS
	FDL	0.622	NS	NS	NS
	ADL	0.397	NS	NS	NS

*FDW* Full dentition width,* ADW* Anterior dentition width, *FDL* Full dentition length, *ADL* Anterior dentition length, *NS* Non-signifcant diference, *P P* value for comparing among three techniques, *P1 P* value for comparing between the control and test group, *P2 P* value for comparing between the control and expansion group, *P3 P* value for comparing between the test and expansion group

*Statistical significance at P ≤ 0.05

**Statistical significance at P ≤ 0.01

***Statistical significance at P ≤ 0.001

**Fig. 3:**
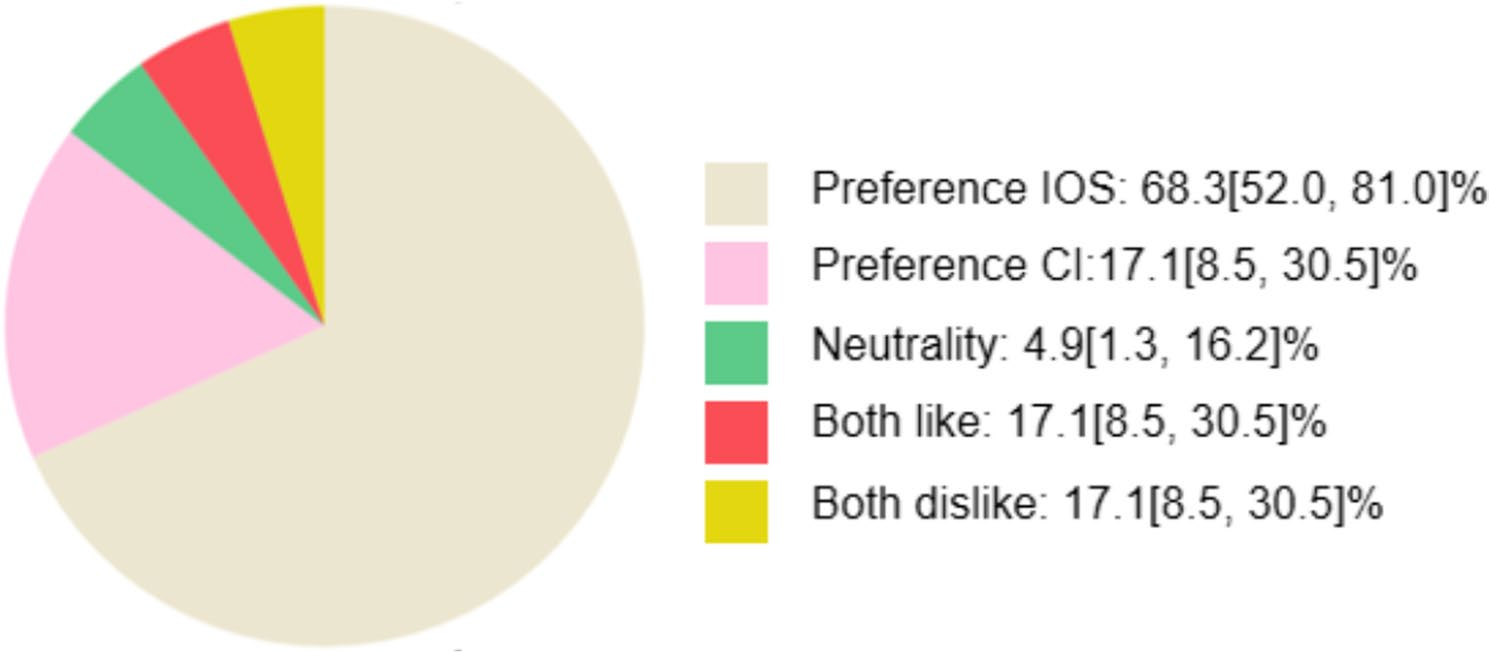
The percentage and 95% confidence interval of preference between IOS and CI. IOS: Intraoral scanner; CI: Conventional impression

The questionnaire results indicated different overall perceptions between IOS and CI (*P* = 0.014), CI was associated with significantly greater queasiness and breathing difficulty (*P* < 0.001), while IOS was more frequently perceived as having an overly large size (*P* < 0.001) (Table 3). There was no significant gender- or age-based differences in the scores for any of the question responses (Supplyment Table 2). Notably, 68.3% [52.0%, 81.0%] of the children preferred IOS compared to only 17.1% [8.5%, 30.5%] for CI, reflecting a clear preference for the digital approach (Fig. 3).

**Table 3:** Descriptive statistics and Wilcoxon Signed-Rank Test results of the questionnaire

	**IOS**		**CI**		***P***
	**Mean(SD)**	**Median (IQR)**	**Mean(SD)**	**Median (IQR)**	
Queasiness	4.12 ± 1.17	5(1)	2.20 ± 1.36	2(3)	<0.001***
Pain	4.15 ± 1.33	5(2)	4.10 ± 1.24	5(2)	0.678
Discomfort when mouth open	3.83 ± 1.46	5(3)	3.61 ± 1.51	4(3)	0.508
Dry mouth	3.46 ± 1.69	5(3)	3.76 ± 1.64	5(3)	0.339
Breathing difficulty	3.90 ± 1.14	4(2)	2.80 ± 1.33	3(2.5)	<0.001***
Nervous or afraid	4.02 ± 1.39	5(2.5)	3.98 ± 1.49	5(2.5)	0.906
Tool with large size	2.12 ± 1.47	1(2.5)	3.32 ± 1.57	4(3)	<0.001***
Take a long time	3.54 ± 1.14	3(2)	3.22 ± 1.49	4(3)	0.307
Total	29.15 ± 5.88	28(9)	26.98 ± 6.53	29(8)	0.014*

*IOS* Intraoral scanner, *CI* Conventional impression, *SD* Standard deviation, *IQR* Interquartile ranges;

*Statistical significance at P ≤ 0.05

***Statistical significance at P ≤ 0.001

## Discussion

This study evaluated the accuracy of IOS and patient satisfaction in children with primary dentition. Using iTero scanning for both intraoral and extraoral settings, and comparing them against high-precision desktop scans. The accuracy assessment incorporated RMS analysis and intra-arch distances, aligning with recommendations to combine surface-based and linear metrics for comprehensive evaluation [28]. At the same time, high reliability surveys were used to collect participants’ perceptions of IOS and CI. To the best of the authors’ knowledge, this is the first approach to compare the accuracy of IOS in primary dentition carried out both in vitro and in vivo using the same reference object. Consequently, the influence of the in vivo environment could be investigated. In addition, this is the first approach to investigate the intraoral scanning satisfaction of preschool-aged children.

The trueness of intraoral scans in our study (0.1282 mm maxilla; 0.1341 mm mandible), well within the 0.5 mm accuracy threshold required for orthodontic applications [29]. Although statistically significant differences were observed in the maxillary ADW and mandibular FDW, the actual mean differences were all below the established clinical threshold, and the corresponding effect sizes were all below the ‘small’ benchmark, indicating no practical impact on diagnosis or treatment. Therefore, we confirm that the trueness of IOS meets clinical requirements. The trueness of IOS in primary dentition was notably higher than that reported in permanent dentition using the same scanner [30]. The dental arch span affects scanning accuracy [7, 23], and the primary dentition has fewer teeth and a smaller dental arch span, which may reduce the computational demand for image alignment and thus contributes to higher trueness. However, the accuracy of TRIOS scanner for mixed dentition in vivo was 0.022 mm [31]. These findings underscore the influence of scanner type on accuracy [7, 32]. Interdental spacing has been shown to negatively impact scan precision in both primary and permanent dentitions [33]– [34], with particular difficulties in capturing occlusal pits, fissures, interproximal surfaces, and preparation margins. The physiological spacing of primary dentition may affect the accuracy of the scan. The superior accuracy of extraoral scanning over IOS for maxillary and mandibular plaster models is in line with earlier findings [7, 10, 35]. Moreover, some studies have reported that extraoral scanning via the iTero Element provides greater accuracy than does the D250 desktop scanner for plaster model [14]. Notably, IOS can also be used to directly scan plaster models, enhancing their versatility and clinical utility.

Our study demonstrated that IOS is the better favored impression method. The subjects preferred IOS (68.3%) over the alginate impressions (17.1%), primarily because of the comfortable feelings and lower level of queasiness. These results are in line with those of previous studies in adults and adolescents [3–6, 36]. Similarly, no significant breathing difficulty was reported in our study, which aligns with the observations of the two studies [3, 5]. No significant pain was noted with either method, likely due to appropriate tray selection and standardized protocols. The high acceptability of IOS contributes to a more positive dental visit experience, reduces dental anxiety, and facilitates subsequent treatment procedures. In contrast to CI, which are often difficult to obtain accurate and standardized impression once with uncooperative children, IOS may be paused to improve accuracy and reduce chairside time. Operation time remains a variable finding across studies. While a systematic review revealed that participants generally perceived IOS as faster than CI [37], Burhardt et al. reported no significant difference [3]. Objective chairside time measurements also varied: Burhardt et al. reported alginate faster than Lava C.O.S [3], whereas Patel et al. reported IOS was significantly faster than alginate [6]. Other studies reported no statistic difference [5, 38]. These discrepancies may result from differences in impression time definitions, operator experience, and patient cooperation. Our study relied solely on children’s subjective responses, which may be limited by their communication ability; thus, incorporating caregiver feedback in future research would increase the validity of patient experience assessments.

The participants reported that the scanning tip was too large. In recent years, some intraoral scanners have introduced options for clinicians to select a scanning tip of appropriate size on the basis of clinical needs. Two studies comparing the accuracy of different tip sizes for scanning permanent dentition [39] and partially edentulous mandible [20] revealed that tip size had a significant impact reduce both trueness and precision. Scanning errors were commonly observed on the distal surfaces of posterior teeth, the proximal surfaces adjacent to prepared teeth, and in areas with interproximal undercuts. A smaller scanning tip can enhance accuracy in these areas, as it allows for more flexible positioning [39]. Additionally, a smaller tip is particularly beneficial for intraoral scanning in patients with limited mouth opening. Research on the accuracy of small-tip IOS for primary dentition is limited, and further studies are needed to balance comfort and precision.

All scans in this study were performed by a single, experienced operator. Although this protocol effectively controlled for inter-operator variability and enhanced data consistency, it may have introduced a specific operator-dependent bias. Previous studies have suggested that repeated use improves IOS accuracy [40]. In pediatric patients, smaller oral spaces and limited tolerance increase the importance of operator skill. Further studies should examine the IOS learning curve in children and explore how training and device design affect performance.

Despite these positive findings, several limitations should be noted. First, only extraoral scan precision was evaluated due to challenges and ethic in repeated intraoral scanning in children, and used plaster model desktop scanning as a reference to evaluate trueness. Second, only one single center IOS system from single-center, and complete primary dentitions were tested, which limit generalizability. Third, despite the implementation of multiple measures to ensure data reliability, the interpretation and generalizability of the questionnaire results may have been influenced by the unquantifiable nature of children’s comprehension and response accuracy, as well as the potential presence of children with undiagnosed or unreported psychological comorbidities. Fourth, this study only included children who could cooperate to complete the impression, but preschool-aged children may show movement or compliance issues, which could affect the accuracy. Additionally, the operation time was assessed subjectively rather than objectively. Future research should aim to enhance the diversity of study subjects, particularly special populations, and incorporate diverse operators with varied skills. Additionally, it is crucial to document objective measurements of operation time. Using dentition direct measurement data as a control and direct assessment of intraoral scan reproducibility to further validate the clinical applicability of IOS in pediatric dentistry. Additionally, further studies comparing different IOS systems, especially newer devices with smaller scanning tips and enhanced features, are needed to validate their accuracy and patient acceptance in primary dentition populations.

## Conclusions

The refined research conclusions demonstrate that skilled operators use the iTero element 2 and strictly adhering to the operating manual can achieve clinically acceptable accuracy of 0.5 mm for complete deciduous dentition in orthodontic applications, although its performance is susceptible to oral environmental factors. Comparative studies revealed that the system exhibits comparable trueness and precision when plaster models are scanned extraorally. It is noteworthy that while preschool-aged children show greater preference for intraoral scanning, the oversized scanning head of iTero remains a significant clinical concern that warrants attention.

## Supplementary Information

Supplementary Material 1.

## Data Availability

No datasets were generated or analysed during the current study.

## Abbreviations

ADL: Anterior Dentition Length
ADW: Anterior Dentition Width
CI: Conventional Impression
FDL: Full Dentition Length
FDW: Full Dentition Width
ICC: Intraclass Correlation Coefficient
IOS: Intraoral Scanner
IQR: Interquartile Range
RMS: Root Mean Square
STL: Standard Tessellation Language

## References

[1] Christensen GJ. Impressions are changing: deciding on conventional, digital or digital plus in-office milling. J Am Dent Assoc. 2009;140(10):1301–4. 10.14219/jada.archive.2009.0054. 19797561 10.14219/jada.archive.2009.0054

[2] Cervino G, Fiorillo L, Herford AS, Laino L, Troiano G, Amoroso G, et al. Alginate materials and dental impression technique: A current state of the Art and application to dental practice. Mar Drugs. 2018;17(1):18. 10.3390/md17010018. 30597945 10.3390/md17010018 PMC6356954

[3] Burhardt L, Livas C, Kerdijk W, van der Meer WJ, Ren Y. Treatment comfort, time perception, and preference for conventional and digital impression techniques: A comparative study in young patients. Am J Orthod Dentofac Orthop. 2016;150(2):261–7. 10.1016/j.ajodo.2015.12.027. 10.1016/j.ajodo.2015.12.027 27476358

[4] Sfondrini MF, Gandini P, Malfatto M, Di Corato F, Trovati F, Scribante A. Computerized casts for orthodontic purpose using Powder-Free intraoral scanners: Accuracy, execution Time, and patient feedback. Biomed Res Int. 2018;2018:4103232. 10.1155/2018/4103232. 29850512 10.1155/2018/4103232 PMC5937598

[5] Yilmaz H, Aydin MN. Digital versus conventional impression method in children: Comfort, preference and time. Int J Paediatr Dent. 2019;29(6):728–35. 10.1111/ipd.12566. 31348834 10.1111/ipd.12566

[6] Patel C, Barot GN, Patel MC, Nath KJ, Patel SP, Patel DK. Accuracy and comfort in digital and conventional impression in pediatric dental patients: A randomized comparative study. Cureus. 2025;17(1):e76882. 10.7759/cureus.76882. 39902027 10.7759/cureus.76882 PMC11788448

[7] Kernen F, Schlager S, Seidel Alvarez V, Mehrhof J, Vach K, Kohal R, et al. Accuracy of intraoral scans: an in vivo study of different scanning devices. J Prosthet Dent. 2022;128(6):1303–9. 10.1016/j.prosdent.2021.03.007. 33902891 10.1016/j.prosdent.2021.03.007

[8] Tallarico M. Computerization and digital workflow in medicine: focus on digital dentistry. Mater (Basel). 2020;13(9):2172. 10.3390/ma13092172. 10.3390/ma13092172 PMC7254335 32397279

[9] Mangano F, Gandolfi A, Luongo G, Logozzo S. Intraoral scanners in dentistry: a review of the current literature. BMC Oral Health. 2017;17(1):149. 10.1186/s12903-017-0442-x. 29233132 10.1186/s12903-017-0442-x PMC5727697

[10] Sun L, Lee JS, Choo HH, Hwang HS, Lee KM. Reproducibility of an intraoral scanner: A comparison between in-vivo and ex-vivo scans. Am J Orthod Dentofac Orthop. 2018;154(2):305–10. 10.1016/j.ajodo.2017.09.022. 10.1016/j.ajodo.2017.09.022 30075932

[11] Ahlholm P, Sipilä K, Vallittu P, Jakonen M, Kotiranta U. Digital versus conventional impressions in fixed prosthodontics: A review. J Prosthodont. 2018;27(1):35–41. 10.1111/jopr.12527. 27483210 10.1111/jopr.12527

[12] Akhlaghian M, Khaledi AA, Farzin M, Pardis S. Vertical marginal fit of zirconia copings fabricated with one direct and three indirect digital scanning techniques. J Prosthet Dent. 2021;126(3):421–6. 10.1016/j.prosdent.2020.03.028. 32868029 10.1016/j.prosdent.2020.03.028

[13] Christopoulou I, Kaklamanos EG, Makrygiannakis MA, Bitsanis I, Perlea P, Tsolakis AI. Intraoral scanners in orthodontics: A critical review. Int J Environ Res Public Health. 2022;19(3). 10.3390/ijerph19031407. 10.3390/ijerph19031407 PMC8834929 35162430

[14] Keul C, Güth JF. Accuracy of full-arch digital impressions: an in vitro and in vivo comparison. Clin Oral Investig. 2020;24(2):735–45. 10.1007/s00784-019-02965-2. 31134345 10.1007/s00784-019-02965-2

[15] Kim J, Park JM, Kim M, Heo SJ, Shin IH, Kim M. Comparison of experience curves between two 3-dimensional intraoral scanners. J Prosthet Dent. 2016;116(2):221–30. 10.1016/j.prosdent.2015.12.018. 27061634 10.1016/j.prosdent.2015.12.018

[16] Revilla-León M, Kois DE, Kois JC. A guide for maximizing the accuracy of intraoral digital scans. Part 1: operator factors. J Esthet Restor Dent. 2023;35(1):230–40. 10.1111/jerd.12985. 36479807 10.1111/jerd.12985

[17] Pattamavilai S, Ongthiemsak C. Accuracy of intraoral scanners in different complete arch scan patterns. J Prosthet Dent. 2024;131(1):155–62. 10.1016/j.prosdent.2021.12.026. 35256181 10.1016/j.prosdent.2021.12.026

[18] Revilla-León M, Subramanian SG, Özcan M, Krishnamurthy VR. Clinical study of the influence of ambient light scanning conditions on the accuracy (Trueness and Precision) of an intraoral scanner. J Prosthodont. 2020;29(2):107–13. 10.1111/jopr.13135. 31860144 10.1111/jopr.13135

[19] Revilla-León M, Gohil A, Barmak AB, Gómez-Polo M, Pérez-Barquero JA, Att W, et al. Influence of ambient temperature changes on intraoral scanning accuracy. J Prosthet Dent. 2023;130(5):755–60. 10.1016/j.prosdent.2022.01.012. 35210107 10.1016/j.prosdent.2022.01.012

[20] Hayama H, Fueki K, Wadachi J, Wakabayashi N. Trueness and precision of digital impressions obtained using an intraoral scanner with different head size in the partially edentulous mandible. J Prosthodont Res. 2018;62(3):347–52. 10.1016/j.jpor.2018.01.003. 29502933 10.1016/j.jpor.2018.01.003

[21] Agustín-Panadero R, Moreno DM, Pérez-Barquero JA, Fernández-Estevan L, Gómez-Polo M, Revilla-León M. Influence of type of restorative materials and surface wetness conditions on intraoral scanning accuracy. J Dent. 2023;134:104521. 10.1016/j.jdent.2023.104521. 37061118 10.1016/j.jdent.2023.104521

[22] Aubreton O, Bajard A, Verney B, Truchetet F. Infrared system for 3D scanning of metallic surfaces. Mach Vis Appl. 2013;24(7):1513–24. 10.1007/s00138-013-0487-z.

[23] Chen Y, Zhai Z, Li H, Yamada S, Matsuoka T, Ono S, et al. Influence of liquid on the tooth surface on the accuracy of intraoral scanners: an in vitro study. J Prosthodont. 2022;31(1):59–64. 10.1111/jopr.13358. 33829613 10.1111/jopr.13358

[24] Son K, Lee KB. Effect of tooth types on the accuracy of dental 3D scanners: an in vitro study. Mater (Basel). 2020;13(7):1744. 10.3390/ma13071744. 10.3390/ma13071744 PMC7178641 32283591

[25] Kaewbuasa N, Ongthiemsak C. Effect of different arch widths on the accuracy of three intraoral scanners. J Adv Prosthodont. 2021;13(4):205–15. 10.4047/jap.2021.13.4.205. 34504672 10.4047/jap.2021.13.4.205 PMC8410304

[26] Serrano-Velasco D, Martín-Vacas A, Paz-Cortés MM, Giovannini G, Cintora-López P, Aragoneses JM. Intraoral scanners in children: evaluation of the patient perception, reliability and reproducibility, and chairside time-A systematic review. Front Pediatr. 2023;11:1213072. 10.3389/fped.2023.1213072. 37435173 10.3389/fped.2023.1213072 PMC10331299

[27] Qian X, Kun Z, Lei W, Jun GAO, Junrun C, Yalan REN. Accuracy of iTero element 1 intraoral scanner digital model in mild to moderate crowding of the maxillary and mandibular arch. J Kunming Med Univ. 2023;44(10):149–54. 10.12259/j.issn.2095-610X.S20231025.

[28] Kihara H, Hatakeyama W, Komine F, Takafuji K, Takahashi T, Yokota J, et al. Accuracy and practicality of intraoral scanner in dentistry: A literature review. J Prosthodont Res. 2020;64(2):109–13. 10.1016/j.jpor.2019.07.010. 31474576 10.1016/j.jpor.2019.07.010

[29] Cuperus AM, Harms MC, Rangel FA, Bronkhorst EM, Schols JG, Breuning KH. Dental models made with an intraoral scanner: a validation study. Am J Orthod Dentofac Orthop. 2012;142(3):308–13. 10.1016/j.ajodo.2012.03.031. 10.1016/j.ajodo.2012.03.031 22920696

[30] de Freitas BN, Capel CP, Vieira MA, Barbin GF, Cardoso L, Tirapelli C. Do intraoral scanning technologies affect the trueness of dental arches with crowding, diastema, and edentulous spaces? A clinical perspective. J Dent. 2024;149:105285. 10.1016/j.jdent.2024.105285. 39103077 10.1016/j.jdent.2024.105285

[31] Liczmanski K, Stamm T, Sauerland C, Blanck-Lubarsch M. Accuracy of intraoral scans in the mixed dentition: a prospective non-randomized comparative clinical trial. Head Face Med. 2020;16(1):11. 10.1186/s13005-020-00222-6. 32430023 10.1186/s13005-020-00222-6 PMC7236363

[32] Amornvit P, Rokaya D, Sanohkan S. Comparison of accuracy of current ten intraoral scanners. Biomed Res Int. 2021;2021:2673040. 10.1155/2021/2673040. 34552983 10.1155/2021/2673040 PMC8452395

[33] Pan CY, Chen MY, Liu CT, Kai-Chun C, Chen JH, Hung CC, et al. Trueness comparison of intraoral scans for diverse arch lengths in pediatric dental models. J Dent Sci. 2024;19(Suppl 2):S149–55. 10.1016/j.jds.2024.07.041. 39807257 10.1016/j.jds.2024.07.041 PMC11725080

[34] Huang MY, Son K, Lee KB. Effect of distance between the abutment and the adjacent teeth on intraoral scanning: an in vitro study. J Prosthet Dent. 2021;125(6):911–7. 10.1016/j.prosdent.2020.02.034. 32473732 10.1016/j.prosdent.2020.02.034

[35] Ye JR, Park SH, Lee H, Hong SJ, Chae YK, Lee KE, et al. Influence of limited mouth opening in children on intraoral scanning accuracy: an in vitro study. Int J Paediatr Dent. 2024;34(6):755–63. 10.1111/ipd.13175. 38480519 10.1111/ipd.13175

[36] Burzynski JA, Firestone AR, Beck FM, Fields HW. Jr.,Deguchi T. Comparison of digital intraoral scanners and alginate impressions: time and patient satisfaction. Am J Orthod Dentofac Orthop. 2018;153(4):534–41. 10.1016/j.ajodo.2017.08.017. 10.1016/j.ajodo.2017.08.017 29602345

[37] Siqueira R, Galli M, Chen Z, Mendonça G, Meirelles L, Wang HL, et al. Intraoral scanning reduces procedure time and improves patient comfort in fixed prosthodontics and implant dentistry: a systematic review. Clin Oral Investig. 2021;25(12):6517–31. 10.1007/s00784-021-04157-3. 34568955 10.1007/s00784-021-04157-3 PMC8475874

[38] Glisic O, Hoejbjerre L, Sonnesen L. A comparison of patient experience, chair-side time, accuracy of dental arch measurements and costs of acquisition of dental models. Angle Orthod. 2019;89(6):868–75. 10.2319/020619-84.1. 31259615 10.2319/020619-84.1 PMC8109162

[39] An H, Langas EE, Gill AS. Effect of scanning speed, scanning pattern, and tip size on the accuracy of intraoral digital scans. J Prosthet Dent. 2024;131(6):1160–7. 10.1016/j.prosdent.2022.05.005. 35738926 10.1016/j.prosdent.2022.05.005

[40] Thomas AA, Jain RK. Influence of operator experience on scanning time and accuracy with two different intraoral Scanners - A prospective clinical trial. Turk J Orthod. 2023;36(1):10–4. 10.4274/TurkJOrthod.2022.2021.0220. 36960781 10.4274/TurkJOrthod.2022.2021.0220 PMC10140657

