# Supplementary material for: Accuracy and patient satisfaction of intraoral scanner in preschool-aged children: a combined in vivo and in vitro study
Source: BMC Oral Health. 2025 Nov 28;25:2001. doi: 10.1186/s12903-025-07406-z (PMC12751212; doi:10.1186/s12903-025-07406-z)
Supplement: Supplementary file 1 — Supplementary Material 1. [file 12903_2025_7406_MOESM1_ESM.docx]

| **Table1. Intra- and interobserver reliability of RMS and intra-arch distances.** |
| --- |
| \|  \| **RMS** \| **FDW** \| **ADW** \| **FDL** \| **ADL** \| \| --- \| --- \| --- \| --- \| --- \| --- \| \| Intra-rater reliability \| 0.993  [0.975, 0.999] \| 0.995  [0.987, 0.999] \| 0.999  [0.998, 1.000] \| 0.964  [0.904, 0.994] \| 0.855  [0.670, 0.974] \| \| Inter-rater reliability \| 0.999  [0.996, 1.000] \| 0.994  [0.975, 0.999] \| 0.999  [0.996, 1.000] \| 0.926  [0.729, 0.988] \| 0.850  [0.521, 0.975] \| |
| RMS: Root Mean Square; FDW: Full dentition width; ADW: Anterior dentition width; FDL: Full dentition length; ADL: Anterior dentition length. |

| **Table2. Results of the gender and age differences test in the survey questionnaire.** | | | |
| --- | --- | --- | --- |
|  |  | ***P*1（Gender）** | ***P*2 (Age）** |
| Queasiness | IOS | 0.832 | 0.600 |
|  | CI | 0.877 | 0.595 |
| Pain | IOS | 0.185 | 0.610 |
|  | CI | 0.342 | 0.250 |
| Discomfort when mouth open | IOS | 0.150 | 0.380 |
|  | CI | 0.328 | 0.501 |
| Dry mouth | IOS | 0.506 | 0.357 |
|  | CI | 0.966 | 0.390 |
| Breathing difficulty | IOS | 0.342 | 0.723 |
|  | CI | 0.372 | 0.073 |
| Nervous or afraid | IOS | 0.150 | 0.296 |
|  | CI | 0.724 | 0.804 |
| Tool with large size | IOS | 0.205 | 0.112 |
|  | CI | 0.250 | 0.220 |
| Take a long time | IOS | 0.314 | 0.414 |
|  | CI | 0.854 | 0.565 |
| Total | IOS | 0.064 | 0.373 |
|  | CI | 0.238 | 0.172 |
| IOS: Intraoral scanner; CI: Conventional impression; *P*1: *P* value for gender difference with Mann-Whitney U test; *P*2: *P* value for age difference with Kruskal-Wallis H test. | | | |
